# Application of CO_2_ waveform in the alveolar recruitment maneuvers of hypoxemic patients during one-lung ventilation

**DOI:** 10.1097/MD.0000000000003900

**Published:** 2016-06-17

**Authors:** Chunshan Dong, Junma Yu, Qi Liu, Chao Wu, Yao Lu

**Affiliations:** a Department of Anesthesiology, Third Affiliated Hospital of Anhui Medical University, Hefei, Anhui Province, P.R. China; b Department of Anesthesiology, First Affiliated Hospital of Anhui Medical University, Hefei, Anhui Province, P.R. China.

**Keywords:** alveolar recruitment maneuver, CO_2_ expirogram, hypoxemic, one-lung ventilation

## Abstract

Deterioration of gas exchange during one-lung ventilation (OLV) is caused by both total collapse of the nondependent lung and partial collapse of the dependent lung. Alveolar recruitment maneuver improves lung function during general anesthesia. The objective of this study was to investigate whether there is an indirect relationship between the changes of CO_2_ expirogram and the selective lung recruitment. To further improve the oxygenation and gas exchange, we compare adjust setting of ventilated parameters based on CO_2_ expirogram and a preset setting of ventilated parameters during OLV in patients undergoing right-side thoracic surgery.

Thirty patients met the requirements criteria that were studied at 3 time points: during two-lung ventilation (TLV), during OLV with preset ventilation parameters (OLV-PP), and during OLV with adjustable ventilation parameters (OLV-AP) that are in accordance with CO_2_ expirogram. Adjustable ventilation parameters such as tidal volume (VT), respiratory rate (RR), positive end-expiratory pressure (PEEP), and the ratio of inspiratory to expiratory were adjusted by utilizing the phase III slopes of CO_2_ expirogram, which together with the relationship between the changes of CO_2_ expirogram and the selective lung recruitment.

During OLV, the phase III slopes of CO_2_ expirogram in patients with pulse oxymetry (SpO_2_) decreased less than 93% after the OLV-PP, and were absolutely different from that during TLV. After OLV-AP, the phase III slopes of CO_2_ expirogram and SpO_2_ were similar to those during TLV. During OLV, however, parameters of ventilation setting in both OLV-PP and OLV-AP are obviously different.

This study indicates that alveolar recruitment by utilizing CO_2_ expirogram probably improves SpO_2_ level during one-lung ventilation.

## 1 Introduction

During lung operations or in instances when the collapse of the lung increases access to the operation field, one-lung ventilation (OLV) is almost always now in thoracic operations. Although nonventilated lung leads inevitably to transpulmonary shunting, and, occasionally, to hypoxemia, not all cases of hypoxemia are altered by shunt and uneven ventilation perfusion ratios during OLV. In patients undergoing general anesthesia, 1 study showed that lung recruitment maneuvers were both easy to perform and effective in reverting alveolar collapse, hypoxemia, and decreased compliance.^[1]^ Hypoxemia during OLV is associated with operation side.^[2]^ Schwarzkopf et al^[3]^ showed the mean arterial oxygen tension during OLV was respectively around 280 and 170 mm Hg during left-sided thoracic surgery and right-sided operation under ventilation with a fraction of inspired oxygen (FiO_2_) of 1. Therefore, oxygenation during OLV is better during left thoracotomy, since the right lung is larger than the left one.^[4]^ Moreover, OLV anesthesia in the lateral position leads to total collapse of the nondependent lung and increase of dead space in the dependent lung, which results in pulmonary shunt ranging from 15% to 40% and atelectasis due to impaired arterial oxygenation, respectively.^[5]^ Therefore, gas exchange and ventilation efficiency during general anesthesia can be evaluated by the analysis of lung recruitment maneuvers and lung CO_2_ removal, as shunt is closely associated with dead space.

Selective recruitment of a collapsed lung lobe on gas exchange and lung efficiency during OLV by using the single-breath test of CO_2_.^[6]^ Schulz et al^[7]^ confirmed that although clear asymmetries in lung structure exist, filling and emptying of the lungs occurs in a remarkably symmetrical fashion. Further, the lungs are robust to changes in ventilatory patterns. The dynamics on the intrapulmonary gas transport is not yet clear because comprehensive studies are lacking. Thus, it is important to provide for further recruitment maneuver to keep the alveoli opened and avoiding uniform alveolar distension. Our study is focused on allowing a more comprehensive characterization of the single-breath expirogram than does the consideration of dead space volumes alone during OLV undergoing thoracic surgeries. The aim of this study was to evaluate the efficacy of alveolar recruitment maneuver followed by utilizing the shape of a carbon dioxide expirogram that is altered with different setting parameters of mechanical ventilation and as a label for gas changes during OLV.

## 2 Methods

This study was approved by the Ethics Board of Affiliated Third Hospital of Anhui Medical University. Between January 2014 and October 2014, consecutive patients scheduled for undergoing elective thoracic surgical procedures (right lobectomy or esophageal neoplasia resection) requiring left one lung ventilation (OLV) and have developed an episode from intermittent droping in pulse oxymetry (SpO_2_) less than 90% appeared 2 times after initial OLV were considered for this study if they met the requirements of the experimental protocol. Written informed consent was obtained from all patients. All patients were enrolled in the prospective longitudinal study and were selected on the following criteria: preoperative evaluation major included a physical examination, patients underwent pulmonary function tests and blood gases, cardiac evaluation (and echocardiography if ordered by the cardiologist), and cardiorespiratory polygraphy if acute respiratory distress syndrome or severe threatened by the presence of a right-to-left transpulmonary shunt were suspected. Exclusion criteria were documented any cardiovascular disease, hypertension or arrhythmia, or major obstructive or restrictive pulmonary disease (defined as <70% of predicted values for pulmonary function test variables of volume and flow), and anemic (hemoglobin <9 g/dL), or liver and renal dysfunction, or inability to maintain an appropriate SpO_2_ or end-tidal carbon dioxide partial pressure (P_ET_CO_2_). In addition, patients who required absolutely right-sided double-lumen tube and presented a distorted anatomy of the tracheobronchial tree on chest radiograph were not included in this study.

No premedication was given. Before the induction of anesthesia, an IV infusion of normal Ringer lactate was started. After 3 minutes of preoxygenation, anesthesia was induced with midazolam 0.1 mg/kg, propofol 1.5 mg/kg, fentanyl 2 μg/kg, and vecuronium 0.08 mg/kg IV and isoflurane concentrations up to 1 MAC. The trachea and the left bronchus were intubated with a left-sided double-lumen tube (DLT) of the appropriate size, and then it was connected to the anesthesia circuit. After clinical confirmation of correct DLT (by inspection and auscultation) with the patient in both the supin and lateral decubitus positions. Effective lung isolation was confirmed by the absence of a leak from the nonventilated lumen of the endobronchial tube. Upon opening of the pleura, direct observation of the collapsed nonventilated lung and the absence of a leak from this lung provided further confirmation. Anesthesia was maintained with low concentration isoflurane (≦1%), and propofol (75 mg/kg/min) and remifentanil (0.1 mg/kg/min). Vecuronium (0.03 mg/kg) was administrated if required muscular relaxing.

All patients were ventilated with Datex-Ohmeda Aestive/5 Smart Ventilator (Madison, WI). Patients were randomized to receive one of the following ventilatory regimens. Before starting OLV, two lung ventilation (TLV) started with 100% FiO_2_, VT of 8 mL/kg predicted body weight, positive end-expiratory pressure (PEEP) of 0 cmH_2_O, inspiratory to expiratory (I:E) ratio of 1: 2, and initial respiratory rate (RR) of 12 breaths/min. After starting OLV, ventilation parameters were fixed at 6 mL/kg VT under 100% FiO_2_, a 14 to 16 breaths/min RR to keep P_ET_CO_2_ between 30 and 35 mm Hg, I: E was 1: 2, and a 6 to 10 cm H_2_O PEEP.

**Figure 1:**
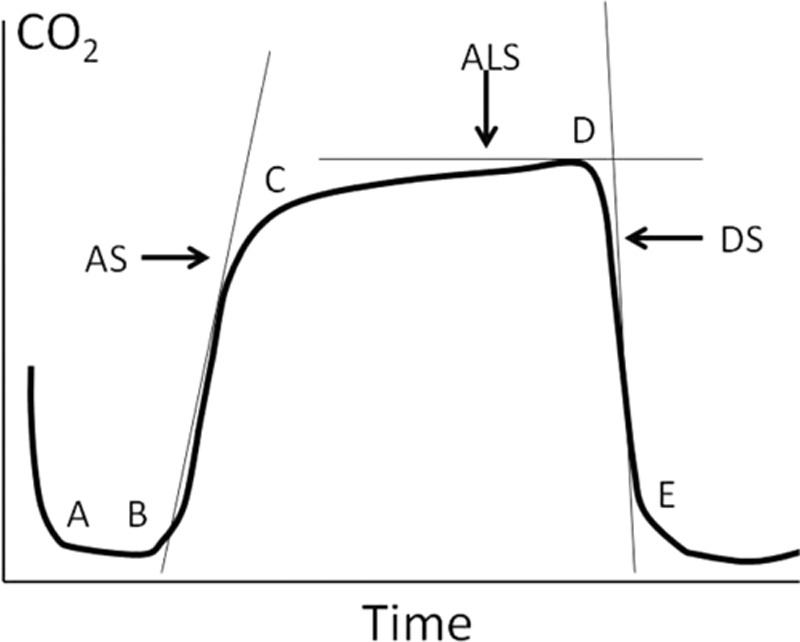
CO_2_ waveform with superimposed waveform parameters. Phase I, points B to C (ascending phase); Phase II, points C to D (alveolar plateau); Phase III, points D to E (descending phase); ALS, alveolar slope; AS, ascending slope; DS, descending slope.

Standard monitoring including electrocardiogram, heart rate (HR), invasive arterial blood pressure, and SpO_2_ was collected by the Datex Ohmeda S/5 monitor during the entire study period. Prior to use, the P_ET_CO_2_ was measured using an infrared analyzer with a side stream sampler attached at the elbow between the endotracheal tube and the anesthesia circuit and the device was calibrated according to the manufacturer's recommendations. The CO_2_ waveform was obtained by monitoring P_ET_CO_2_ on a monitor following tracheal cannulation during the two lung ventilation and as a basic CO_2_ waveform. However, CO_2_ waveforms are characterized by a triphasic shape that has been described in normal (see Fig. 1),^[8]^ therefore it is referenced as a basic CO_2_ waveform during TLV.

The primary target variable of this study was CO_2_ waveform by monitoring P_ET_CO_2_. The null hypothesis was that CO_2_ waveform of TLV and OLV modes was propinquity and the alternative hypothesis that they were different. The primary outcome variable was determined by whether arterial oxygenation. (≤93%) once again after restarting OLV (9.6 ± 2.7 min) with PP setting. The patients were carried out by randomization and step-wise approach in both the preset parameters (OLV-PP) setting and adjustable parameters (OLV-AP) setting to the management of arterial oxygenation during OLV. In the OLV-PP setting, ventilation parameters were fixed as described above for an initial control period during OLV. In the OLV-AP setting, the same 100% FiO_2_ was used, the ventilation parameters were randomly adjusted any at a time to following CO_2_ waveform aiming to make its sample with basic CO_2_ waveform as similar as possible. Primary outcome variable was SpO_2_ during TLV, OLV, and period after OLV-AP setting. VT, RR, PEEP, I:E, peak inspiratory pressure (PIP), and P_ET_CO_2_ during mechanical ventilation were recorded. Seven time points were observed and recorded according to the SpO_2_ change. SpO_2_ were a typical normal by TLV and a time point of OLV beginning (*T*_0_). And the processing of hypoxemia included the first time when SpO_2_ decreased to less than 90% (*T*_1_), surgery was temporarily interrupted to resume TLV (performed with continuous positive airway pressure (CPAP)) using the hand bag until SpO_2_ recovered to at least 97% (*T*_2_), once again SpO_2_ decreased to less than 93% after restarting OLV (*T*_3_); SpO_2_ recovered to more than 97% with second attempts of CPAP and AP setting was adopted (*T*_4_), a period after OLV-AP setting was adopted (*T*_5_), and the end of OLV (*T*_6_). That is, OLV-PP setting for *T*_1_, *T*_2_, *T*_3_, and OLV-AP setting for *T*_4_, *T*_5_ and *T*_6_. VT, RR, I:E, PEEP, PIP, and P_ET_CO_2_ were recorded continuously. At frequent intervals, a mean value of all SpO_2_ was obtained during OLV.

## 3 Statistical analysis

Data are presented as mean standard deviation and range. Student *t* test was applied for statistical comparisons of changes occurring within each study condition. A *P* value of <0.05 was considered to achieve statistical significance.

## 4 Results

Thirty patients who met the requirements criteria were included in the study, their physical status I or II, aged 43 to 75 years, and patients characteristics are listed in Table 1.

**Table 1:**
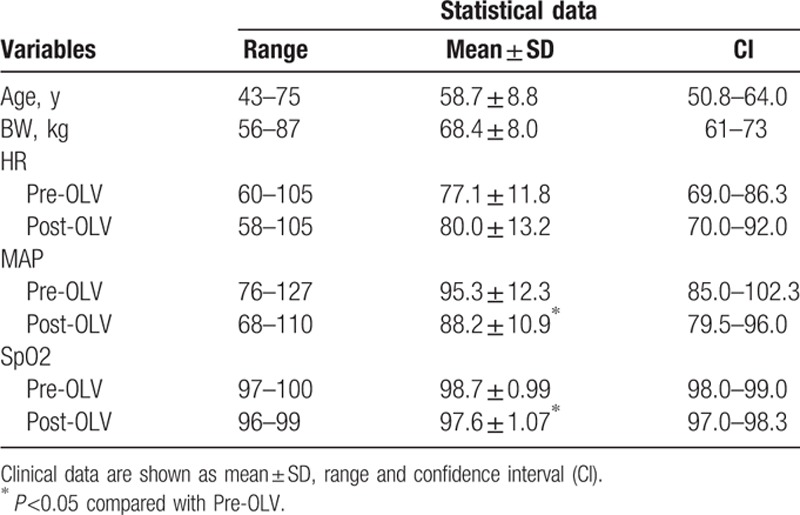
Clinical data collection from all patients in the study.

Thirty patients were presented a SpO_2_ decrease less than 90% (as cases of an episode of hypoxemia) during OLV and randomized or step-wise to receive ventilation parameters with both OLV-PP setting and OLV-AP setting. No patient was excluded from 30 patients due to any preoperative or intraoperative criteria, and in all patients left-double lumen tubes were used.

**Figure 2:**
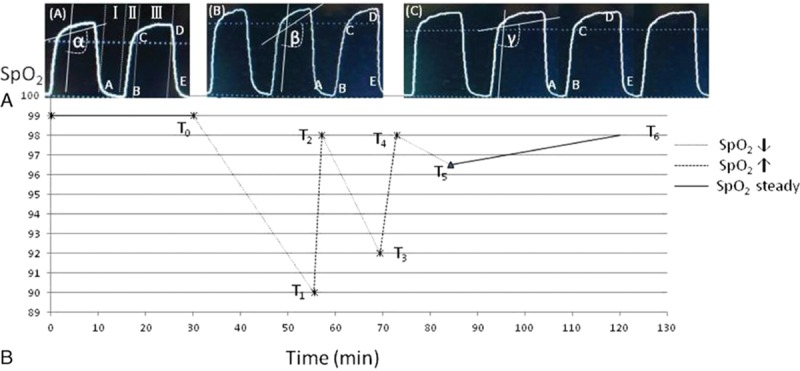
Upper panel (A): the CO_2_ waveform was obtained from an adult patient by the real-time monitor. Note the qualitative difference in the Phase II segment of this CO_2_ waveform when compared with that of differences between the different parameters setting during L-OLV and TLV. (a)-(A) Capnogram, P_ET_CO_2_ was plotted against time (sec) for 1 single-breath during TLV (as q.v. basic waveform). (a)-(B) Capnogram, after application of OLV, displays actual difference of an expired breath during OLV, the slope of the alveolar plateau (angle β vs angle α) was steeped. (a)-(C) Capnogram, by setting trapezoid shape similar to (a)-(A). Lower panel (B): displays actual time point of relevance between the SpO_2_ change and the intraoperative during mechanical ventilation with TLV and L-OLV or was performed with CPAP to both lung using the hand bag.

Fig. 2a (a real sample) was obtained from a patient undergoing OLV and TLV during thoracic surgery. (a)-(A) During TLV, rising segment (phase II) rapidly reaches a height, usually attained only CO_2_ exhaled from rapidly emptying alveoli, whereas alveolar plateau (phase III) would be nearly horizontal. However, this ideal situation does not occur, even in normal lungs. The waveform analysis was performed on theirs difference as the intersection angle between lines B to C (phase II) and lines C to D (phase III) along with principle axis. The beginning of OLV with PP setting depicted a CO_2_ waveform. Fig. 2a-(B) shows that application of PP setting increased the intersection angle between phase II and phase III slopes [beta angle (β = 130°) vs. alpha angle (α = 105°)]. Application of AP setting significantly decreased the angle between phase II and phase III slopes [gamma angle (γ = 110°) vs. beta angle (β = 130°)]. In other words, phase II rapidly reaches a height, whereas phase III would be nearly horizontal, and there is a rapid S-shaped upstroke on the tracing due to the CO_2_ rich exhalation from the alveoli.

In Fig. 2b, the plot shows the entire sequence from intraoperative in SpO_2_ less than 90% in 30 patients during TLV and OLV in accordance with a sample CO_2_ waveform at below 7 time points.

The period before *T*_0_ indicates that SpO_2_ were a typical normal (≥99%) by TLV.

*T*_0_ to *T*_1_ indicates that SpO_2_ were declined (≤90%) of short period (18.5±3.7 min) by OLV according to the PP setting. *T*_1_ to *T*_2_ indicates that SpO_2_ were rose rapidly (2.1 ± 1.5 min) by TLV with CPAP. *T*_2_ to *T*_3_ indicates that SpO_2_ decreased (≤93%) once again after were restarted OLV (9.6 ± 2.7 min) with PP setting. *T*_3_ to *T*_4_ indicates that SpO_2_ were rose again by TLV (2.2 ± 1.8 min) with CPAP. *T*_4_ to *T*_5_ indicates that SpO_2_ were declined briefly by OLV (8.3 ± 3.2 min) with AP setting. *T*_5_ to *T*_6_ indicates that SpO_2_ can be retained normal level by AP setting during OLV (117.6 ± 23.7 min).

Table 2 shows the key characteristics of each setting. They were significantly different with respect to VT, RR, I:E, PEEP, PIP, and P_ET_CO_2_. Arterial oxygenation (SpO_2_) with a mode of the OLV-AP setting was significantly improved compared to the OLV-PP setting. The beginning of OLV with OLV-PP setting produced a significant high in RR and end-tidal carbon dioxide partial pressure (P_ET_CO_2_), whereas comparison of the OLV-PP and OLV-AP showed a significant difference in VT, I:E, PEEP, and PIP.

**Table 2:**
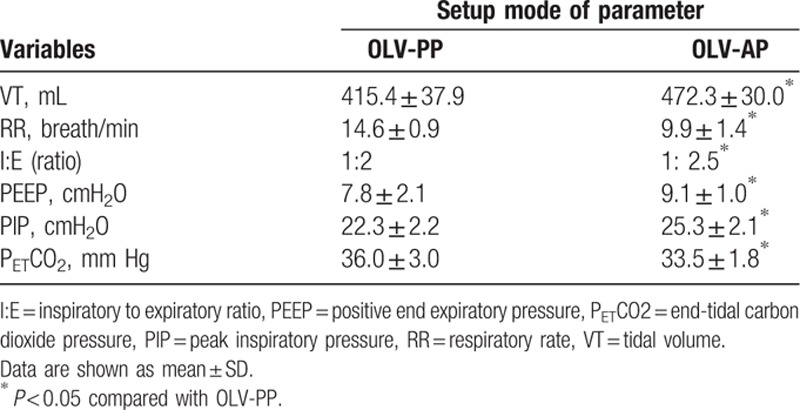
Comparison of variable value between preset parameters setting (PP) and adjustable parameters (AP) setting for patients during one-lung ventilation.

## 5 Discussion

The ventilatory pattern should be directed toward minimizing dynamic hyperinflation and auto-PEEP by using small VT and preserving expiratory time.^[9]^ Although hyperinflation is unlikely with low VT, especially during high FiO_2_, it may lead to more atelectasis and poor oxygenation.^[10]^ Therefore, low VT must be applied with PEEP to avoid atelectasis. We ventilated patients undergoing left-side OLV for right-side thoracic surgery with a low VT as a fixing parameters setting and found that it was difficulty maintaining arterial oxygenation or decrease the occurrence of SpO_2_ less than 90%. In contrast, ventilating patients with selectable lung recruitment maneuver by some transform parameters that mainly based on the change with “CO_2_ expirogram” approach, results in improving the efficiency on oxygenation and gas exchange.

Lung recruitment maneuver has been considered the conventional approach to mechanical ventilation of patients undergoing thoracic surgery and OLV. However, the collapsed lungs could be reexpanded via various strategies during general anesthesia. Atelectatic lung can be completely reexpanded just with 15 seconds of an airway pressure of 40 cm H_2_O. This pressure is corresponding to inflation to vital capacity, and therefore this maneuver is termed vital capacity maneuver.^[11]^ Talab et al^[8]^ demonstrated this maneuver requires to be maintained by PEEP 10 cm H_2_O with the purpose of reexpanding all previously collapsed lung tissues. But during OLV, the recruitment effect of a single vital capacity maneuver may be lost after reduced tidal volumes (5–6 mL/kg) or may be decreased by the intrinsic PEEP because of the accompanying need for increased respiratory rates.^[12]^ For this reason, we believe that as a net result, recruitment maneuvers should protect collapse-prone lungs despite an increase in VT at a slight high PIP. Selective recruitment of the collapsed lung region by adjusting the parameters of ventilation constant only in CO_2_ expirogram for reference never reported to be effective as a last resort for oxygenation of the collapsed lungs.

The SpO_2_ did not decrease less than 97% in any of the 30 patients during TLV with a FiO_2_ of 1.0 in this study. Since mean difference between SpO_2_ and O_2_Hb during OLV was 2.9% ± 0.1%, and the SpO_2_ to O_2_Hb difference decreased with increasing FiO_2_.^[3]^ The SpO_2_ decreased less than 90% is defined as hypoxemia in this study. However, the oxygenation of patients is affected also by the duration of OLV, being the lowest at about 27 minutes with a peak at about 1 hour and 25 minutes.^[13]^ To minimize the impact of this intraoperative variation in oxygenation,^[14]^ we used the 3 stepwise sequence for SpO_2_ decreased less than 93% at 2 times in random order. Although we tried to maintain the parameters of ventilation constant as a preset approach during initial OLV, we had to use an interference of continuous positive pressure (using hand bag) to recover SpO_2_ with oxygenation. This may explain why the improvement in oxygenation with adjustable parameters setting being based on the shape of CO_2_ expirogram was effective in our study.

The time period between the beginning of OLV and applying adjustable parameters setting ranged approximately from 20 to 38 minutes. Our study shows that initial stage during OLV with a predetermined parameter values including VT, RR, I:E, and PEEP, then the graphic of CO_2_ expirogram was correspondingly changed after switching from TLV to OLV. This means quite unlike with other previous results^[6,15–18]^ and can be explained by a recruitment effect on both the function state of small airway or alveolar and dead space, taking into account that hemodynamic, delivered tidal volume, and ventilatory conditions during OLV were different during TLV. This means that graphic changes of CO_2_ expirogram may be an effective means of decreasing oxygenation or affect gas exchange or lead to hypoxemia during OLV. The CO_2_ expirogram (capnogram) has been characterized for adult patients with normal and abnormal pulmonary function,^[19]^ and factors directly related to the airway pressure, the alveolar opening, and ventilation compliance and lung function influence the shape change of a CO_2_ expirogram.^[7,20,21]^ Whether the parameters setting of ventilation according to the shape change of CO_2_ expirogram during the relatively long period of OLV may be “optimize” oxygenation approach is indeed unclear. However, a similar relationship between the shape change of CO_2_ expirogram and the selectable lung recruitment maneuver, and CO_2_ expirogram are significantly more sensitive to the function of the alveolar state than the artificial setting for ventilation parameters in the clinical study. Furthermore, such as application of selectable lung recruitment maneuver by CO_2_ expirogram to the nonventilated lungs, may at least obviate the need for CPAP during OLV.

The results of this study indicate an improved efficiency in gas exchange after a lung recruitment maneuver with ventilation parameters setting by CO_2_ expirogram during OLV. CO_2_ expitogram is a technical monitor of the estimated alveolar PCO_2_ (P_A_CO_2_) and consequently of the estimated arterial PCO_2_ (PaCO_2_) as well as the overall adequacy of alveolar ventilation. In contrast, P_ET_CO_2_ measures the average PACO_2_ accurately only if the volume-weighted alveolar plateau is nearly horizontal on the capnogram. What are the mechanisms that cause or increase the positive slope of the alveolar plateau? Since the chronic obstructive pulmonary disease is accompanied with ineffective ventilated lung units with high PCO_2_, the sequential emptying of parallel alveolar units with different PCO_2_ can generate a sharp slope of alveolar plateau.^[22]^ Our real-time simulation and clinical studies show close relationships between the magnitude of ventilation parameters and the alveolar plateau or the phase III slope. In our study, the VT, PEEP, and PIP after the adjustable parameters setting were higher as compared with preset parameters setting during OLV. This means that a full tidal volume can create a common alveolar opening to the collapsed lungs and constant inspired flow toward the peak inspiratory pressure, and for maximum improvement in oxygenation, continuous PEEP was applied with prior inflation of the lung. We had to use a slow respiratory rate and a setting for I:E ratio of 1 to 2.5 applied by adjustable setting in order to be unanimous in CO_2_ expirogram with one during TLV, which acquiring appropriate expiratory flow process and maintaining the set expiratory pressure (the alveolar plateau of the CO_2_ expirogram will be flatter than applied without prior inflation). Thus, when expiration is prolong and progresses to a lung volume below closing capacity, expired CO_2_ concentration may rise sharply at the end of the alveolar plateau. This may explain why the positive slope of the alveolar plateau is the continued accumulation of CO_2_ from the pulmonary blood into a shrinking alveolar volume during exhalation.^[23]^ The resultant expiratory alteration with flow and pressure had only primary influence on CO_2_ expirogram position and shape of phase II. The general condition of the patient actually represents one of the major factors determining the arterial oxygenation during OLV. Theoretically, however, “optimum” ventilation parameters such as the PEEP, I:E, and PIP may result in more homogeneous distribution of VT, improvement in static and dynamic lung compliance, better oxygenation, and dead space ventilation.

In conclusion, the appropriate lung recruitment improves gas exchange and ventilation efficiency during OLV anesthesia. CO_2_ expirogram by P_ET_CO_2_ monitoring provides an indirect guide of the lung recruitment maneuver gained by ventilation parameters setting. Based on our finding, we believe that CO_2_ expirogram monitoring should be incorporated into “optimize” recruitment approach during OLV and the outcome in connection with ventilation setting to provide insight into the condition of patients suffering hypoxemia.
